# Segmentation-based deep 2D-3D multibranch learning approach for effective hyperspectral image classification

**DOI:** 10.1371/journal.pone.0321559

**Published:** 2025-05-30

**Authors:** Tanver Ahmed, Adiba Mahjabin Nitu, Masud Ibn Afjal, Md Abdulla Al Mamun, Md Palash Uddin

**Affiliations:** 1 Department of Computer Science and Engineering, Hajee Mohammad Danesh Science and Technology University, Dinajpur, Bangladesh; 2 Department of Computer Science and Engineering, Varendra University, Rajshahi, Bangladesh; Universidade Federal de Uberlandia, BRAZIL

## Abstract

Deep learning has revolutionized the classification of land cover objects in hyperspectral images (HSIs), particularly by managing the complex 3D cube structure inherent in HSI data. Despite these advances, challenges such as data redundancy, computational costs, insufficient sample sizes, and the curse of dimensionality persist. Traditional 2D Convolutional Neural Networks (CNNs) struggle to fully leverage the interconnections between spectral bands in HSIs, while 3D CNNs, which capture both spatial and spectral features, require more sophisticated design. To address these issues, we propose a novel multilayered, multi-branched 2D-3D CNN model in this paper that integrates Segmented Principal Component Analysis (SPCA) and the minimum-Redundancy-Maximum-Relevance (mRMR) technique. This approach explores the local structure of the data and ranks features by significance. Our approach then hierarchically processes these features: the shallow branch handles the least significant features, the deep branch processes the most critical features, and the mid branch deals with the remaining features. Experimental results demonstrate that our proposed method outperforms most of the state-of-the-art techniques on the Salinas Scene, University of Pavia, and Indian Pines hyperspectral image datasets achieving 100%, 99.94%, and 99.12% Overall Accuracy respectively.

## 1 Introduction

Hyperspectral Imagery (HSI) has made a pivotal development in the field of remote sensing by combining spectroscopic detail with visual mapping. It provides unparalleled insight into earth objects, as it can capture the spatial layout along with several hundred or thousands of consecutive narrow bands [1–4]. Because of its being highly effective in distinguishing between different types of land cover and surface elements, hyperspectral imaging has gained widespread use in a diverse range of areas, including environmental monitoring, plant ecology, urban development, vegetation analysis, and atmospheric research, among others [5–9]. HSIs offer rich, comprehensive data due to their high resolution and wide spectral range, but their abundance might make analysis more difficult [10].

HSI categorization strives to allocate each pixel vector to a geographic area. There is an enormous challenge to using the abundance of spectral and spatial characteristics in HSIs. The high dimensionality of hyperspectral data causes the Hughes effect, even if the many spectral bands provide potentially useful information [11]. As a result, classification accuracy increases initially before declining as dimensionality rises. One popular approach in HSI classification to address these challenges is to use dimensionality reduction or feature reduction as a first step. By extracting significant features and then choosing a leading subset from these features, feature reduction may be accomplished. Both traditional machine learning (ML)-based techniques and deep learning (DL)-based techniques may be used to extract features and classify hyperspectral images.

Traditional methods for classifying HSIs have often concentrated on spectral data, frequently ignoring the significance of spatial information. While band selection, Principal Component Analysis (PCA) [12], and sparse representation classifiers have been used to find distinguishing features, their feature extraction skills, and general resilience are significantly inadequate. The commonly used ML algorithms, like Support Vector Machine (SVM) [13] and Random Forest (RF) [14], struggle to handle non-linear data and build intrinsic decision boundaries. RF may face difficulties grasping the intricate spatial-spectral relationships. Conversely , SVM may fail to provide decent performance with a high-dimensional vector space.

As mentioned earlier, high-dimensional data creates complexity and redundancy; to overcome these issues, it is crucial to introduce dimensionality reduction as a preprocessing phase. Dimensionality reduction comprises two categories: for HSI band selection and feature extraction. Whether exploiting label information or not, feature extraction methods like linear discriminant analysis (LDA) [15], PCA, kernel PCA (KPCA) [16], and Fisher’s linear discriminant analysis (FLDA) [17] attempt to maximize between-class separation. Guided image extraction using PCA is used in a change detection technique named EPOCH [18], while another approach for image enhancement named PLEASANT exploits gamma correction and PCA [19]. A glacial hypothesis for Martian outflow channels utilizes PCA for extracting maximum variance information [20], and to obtain HSI’s local features, PCA is employed in highly correlated subgroups in [21,22]. Another dimensionality reduction technique named minimum noise fraction (MNF) [23] is useful in specific situations. However, in the case of HSI classification, it may not provide good performance because it assumes that bands and data noise are not correlated. This assumption might not apply to HSI datasets with intricate spatial and spectral relationships [24]. On the other hand, the band selection techniques are capable of identifying vital band groups. Nevertheless, they often failed to grasp the intricate spatial-spectral relationships of HSIs. The effectiveness of well-known feature selection techniques for HSI classification, such as chi-squared, select K best, and mutual information feature selection [25], may be restricted due to the inherent difficulties in capturing the complex spectral and spatial characteristics particular to HSI datasets.

Several Convolutional Neural Network (CNN) design architectures have shown impressive results concurrently exploiting both the spatial and spectral information [26]. The inclusion of DL techniques paves a potential pathway to achieve precise classification rates in HSIs. Computer vision has entered a new era with the introduction of deep learning, which has simplified intricate feature extraction procedures and produced previously unheard-of results in image-related tasks like semantic segmentation and classification. Nevertheless, the initial DL approaches emphasized the spectral information, giving low attention to the spatial layout of the images [27,28]. This leads to the absence of spatial details compromising the classification performance. In recognition of the spatial information, researchers have started to highlight it and address the issue by implementing two-dimensional convolutional neural networks (CNNs) [29,30]. Ingenious structures shown in [30], compress pixels into cubes to record spatial context more effectively, thereby improvising spatial information extraction, while among the other innovative methods, the scientists in [31] utilize pixel similarity for labeling. Pan *et al*. [32] introduce the multi-grained network, a small-scale data-driven approach, whereas the DenseU-Net [33] based on tuna swarm optimization is also a kind of small-scale data-driven technique.

CNNs are excellent at extracting features; by increasing depth and stacking modules, they can learn abstract characteristics that are complicated. One major concern about the deeper network is its increased parameters and more time consumption for computations. GPUs have been created as a solution to these problems, decreasing the training time for large parameter networks. The vanishing gradient issue in deep networks is another obstacle that might make it difficult to learn intricate patterns. Residual networks with skip connections alleviate this issue [34] and are becoming more and more prominent in computer vision, including HSI classification. To enable discriminative feature learning, for instance, Zhong *et al*. [35] constructed a Spectral-Spatial Residual Network (SSRN) containing residual blocks even in the case of a small training dataset. 2D and 3D CNNs are the most common CNNs used in HSI classification methods. A 3D CNN-based model in [36], exploits both spatial and spectral components concurrently with various 3D convolution sizes to provide a model with few parameters, and another approach named Fast and Compact 3D-CNN in [37], implements incremental PCA (IPCA) for preprocessing purposes and is followed by 3D-2D CNN, which reduces spectral-spatial features and classifies HSIs. Spectral Network [38], a wavelet-based structure, derives spatial and spectral information efficiently to categorize HSIs, while Hybrid Spectral Network (HybridSN) provides an architecture combining three 3D CNNs and a 2D CNN [39]. With multi-branch feature fusion, the method exploits the advantages of both 2D and 3D CNNs. This method improves accuracy over conventional CNN-based methods by better combining spectral and spatial information, which strengthens the feature extraction capabilities. Another multibranch feature fusion approach is introduced in [40], all the branches have the same amount of kernels.

CNNs are a type of DL architecture that provides exceptional efficacy in HSI classification by extracting spatial and spectral features efficiently. However, prominent challenges include local minima convergence, potential information loss within pooling layers, and hyperparameter tuning for single-branch networks. While 3D CNN captures joint spectral-spatial elements but may perform poorly when textures have similarities across bands, 2D CNN may overlook interband information. The combination of 3D and 2D CNNs with diverse kernel dimensions serves to improve spatial representation. To overcome these obstacles, we present an HSI classification approach based on 3D-2D CNN and multi-branch feature fusion. To exploit the local intrinsic information, we adopt Segmented PCA (SPCA) as a part of the preprocessing phase. To make a grouping of the features according to their significance, we use minimum Redundancy - Maximum Relevance (mRMR). We construct a neural network consisting of three branches with a combination of 2D CNN and 3D CNN. Extracted features from the branches are carried out to the fully connected layer after fusing. The following is a summary of this paper’s major contributions:

- Our innovative approach combines information from sub-regions inside HSI cubes. This is achieved in the preprocessing step using SPCA and mRMR.
- The shallow-to-deep 3D-2D multi-branch structure of our approach extracts features effectively.
- We thoroughly test our method on three benchmark datasets, demonstrating its wide applicability and offering practical proof of its effectiveness.

The structure of this paper is as follows: Section 2 explores the related theory, and Section 3 provides a comprehensive idea and derivation of our proposed model. Section 4 outlines the experimental setup and provides a comparative analysis of the results. Finally, Section 5 offers a summary of the findings and conclusions drawn from this study.

## 2 Related methodologies

### 2.1 Principal component analysis (PCA)

PCA is broadly adopted to reduce the complexity of HSI data and extract key features. Its primary objective is to transform the multi-dimensional, complex HSI data into a simpler format with fewer dimensions while maintaining the pertinent information. To do this, principal component analysis (PCA) finds the most important patterns in the data, or principal components (PCs), which stand for the directions of highest variability in the dataset. These PCs are perpendicular to one another and are ordered according to how much they contribute to the overall variance in the data [41,42]. Because of its underlying assumptions of linear and orthogonal data distribution, PCA does not succeed in adopting the intricacies of real-world data, ignoring significant nuanced information that is vital for precise classification [43,44]. To consider the local intrinsic information, the PCs are extracted by applying PCA to the highly correlated bands [40,45]. Using a limited number of PCs reduces the complexity of HSI data. This decrease eventually improves the analysis and classification procedures in many HSI applications by helping with visualization, noise reduction, and the extraction of important characteristics. For PCA deployment: let $x_{n}=[x_{n1}x_{n2}...x_{nF}{]}^{T}$ the spectral vector be in the HSI data matrix of a sample pixel, where nε[1,S] and S=X×Y. **D** represents the data matrix has a size of F×S and the spatial domain is indicated by *X* and *Y*. The zero mean image is $I=[I_{1}I_{2}...I_{n}]$ obtained from **D**, where $I_{n}=x_{n}-M$ and $M=\frac{1}{s}\sum_{n=1}^{S}x_{n}$. The eigendecomposition formula is then used to compute the covariance matrix $C=\frac{1}{s}={II}^{T}$, and it is as follows:

$$C={VEV}^{T}.$$ (1)

In this equation, eigenvectors and their associated eigenvalues are denoted by *V* and, *E* respectively. A new matrix **W** is generated from selected *q* eigenvectors, where *q*<<*F*. Sorting the eigenvalues from highest to lowest is the conventional way of determining which *q* main components are the most important. In the last step, a projection matrix **Z = W^T^ × I** is generated from **D**.

### 2.2 Convolutional neural network (CNN)

CNNs are a kind of feedforward neural network. Inside its receptive field, the artificial neuron receives information from a certain area of nearby cells. This particular architecture enables CNNs to process large-scale image data effectively [25,46–48]. To improve HSI classification, Liu *et al*. [49] developed an adaptive multi-feature fusion graph convolution network, while Chen *et al*. [50] established a multiscale superpixelwise random patched fusion network. An efficient HSI classification technique combining spatial and spectral features using network transformation is introduced in [51]. In the same way, a better way to extract features exploiting a 2D-CNN with independent branches for spectral and spatial features is presented in [52].

Two major concepts comprise the CNN architecture. It first exploits the two-dimensional structure of pictures by figuring out that nearby pixels are typically closely connected. The second one is feature sharing. Here, to create a thorough feature map, the network uses convolution to apply the same filter to every area of the image. The convolutional layer, activation function, pooling layer, and fully connected layer are the main components of the CNN.

The convolutional layer’s mathematical operations are carried out using the following formula:

$$X_{i}^{l}=\sum_{j=1}^{f}\Phi ({W_{ji}}^{l}*{X_{j}}^{l-1}+{b_{i}}^{l}),$$ (2)

where (*l*–1)th layer’s feature map is denoted by $X_{i}^{l-1}$, while *l*th layer’s feature map is represented by $X_{i}^{l}$ and *f* refers to the quantity of feature maps. The bias and weight are represented, respectively, by variables ${W_{ji}}^{l}$ and ${b_{i}}^{l}$. Initially, random values are assigned to these parameters. The rectified linear unit (ReLU) function is used in this article, where function Φ stands for a nonlinear activation function. The symbol * signifies the convolutional operation. The pooling layer follows the convolutional layers. The pooling layer’s typical function is to reduce the data’s spatial dimensions. This decrease helps prevent overfitting by reducing the number of network parameters. The fully connected layer, the final component, transmits output data to the classifier by connecting many neurons to the preceding layer. The backpropagation technique is used to train the neural network’s parameters.

CNN enables precise picture extraction by combining convolutional, pooling, and fully connected layers flexibly. The two most common CNN-based HSI classification techniques are 2-D and 3-D CNN. Although combining 2D and 3D CNN designs can improve performance to some extent, it is still difficult to figure out how many network layers are ideal. This is because ineffective feature extraction occurs in networks that are either too deep or too shallow. This research uses a variety of preprocessing approaches in conjunction with 2D and 3D CNNs with different kernel sizes to create a multi-branch architecture that acquires more refined and diversified features.

### 2.3 Feature fusion

Low-level CNN features lack semantics but have vast spatial sizes and comprehensive information. High-level characteristics are less detailed and have a smaller spatial dimension, but they have greater semantics. Exploiting the advantages of both the low-level features and high-level features in a balanced manner and avoiding their limitations improves the model’s performance [53]. Depending on when the fusion and prediction processes take place, feature fusion approaches may be divided into two categories: early fusion and late fusion. In early fusion, multilayer features are first fused, and then a predictor is trained using the fused features. Concatenation or addition procedures are used in this method, which is also known as skip connection. On the other hand, late fusion improves performance by combining several layers’ detection findings. We use concatenation for feature fusion in this study.

## 3 Proposed model

**Fig 1:**
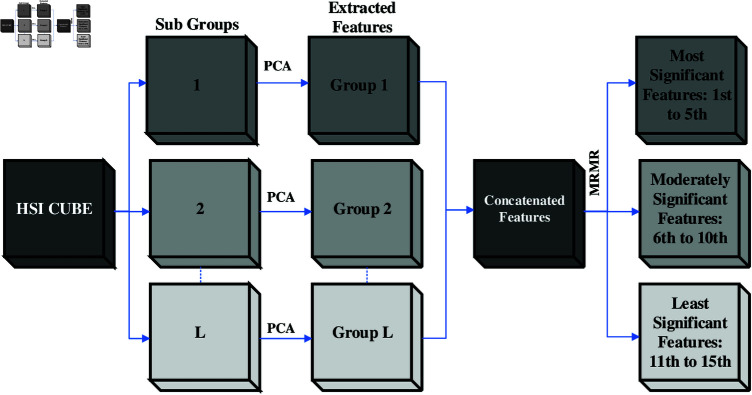
Preprocessing of HSI.

**Fig 2:**
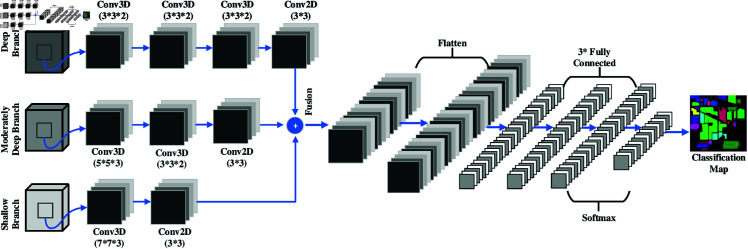
Proposed multibranch CNN architecture.

The suggested approach improves the performance of HSI classification by combining several approaches—SPCA, mRMR, 3D-2D CNN, and multi-branch feature fusion. Images are first preprocessed using mRMR and SPCA. The features are then grouped according to their relevance and fed into a three-branch neural network, which extracts features at various levels. Finally, a softmax layer and fully linked layers are used for categorization. The overall architecture is shown in Figs 1 and 2.

### 3.1 Preprocessing of HSI

To handle highly correlated blocks in HSI data with low inter-correlations more effectively and increase classification accuracy, SPCA is suggested in [17,45,54,55]. First, we use SPCA to HSI in this research to eliminate spectral redundancy in local and global data. The HSI data matrix is initially divided into a *L* subset of datasets for SPCA implementation, based on band correlation analysis. Strongly connected bands are grouped within the same subgroup to represent the local characteristics of the dataset. *L* varies from 2 to 5 in this research. Table 1 shows the details of the segmentation. Subsets of the data matrix ready for SPCA application are defined by the letter $D_{t}$, and tϵ[1,2,3,4,5]. Each split data matrix contains *n*_*t*_ number of bands. For instance, $D_{1}=[I_{1}^{\prime }I_{2}^{\prime }...I_{n_{1}}^{\prime }]$, $D_{2}[I_{1}^{\prime \prime }I_{2}^{\prime \prime }...I_{n_{2}}^{\prime \prime }]$ and so on. Here, the first *n*_1_ rows of the corresponding $I_{j}$ build up the $I_{j}^{\prime }$. Similarly, $I_{j}^{\prime \prime }$ is made out of the next *n*_2_ rows from $I_{j}$ after skipping the first *n*_1_ rows. These processes are performed for every j,nε[1,S]. The projection matrices from each subgroup’s *D*_*t*_ are successively combined to generate the overall projection matrix. Considering D=X×Y×F as an input data matrix, it is converted to projection matrices $Z_{t}=X\times Y\times B_{t}$. *B*_*t*_ is the extracted features from *t*th segment.

**Table 1:** Segmentation details of the datasets: Indian Pines (IP), Salinas Scene (SC), and The Pavia University (PU). Seg-*L* denotes the dataset is split into *L* highly correlated subgroups; in Seg-3, the dataset is split into 3 sub-groups.

Segment no (L)	Parameters	IP			SC			PU	
		Seg-3	Seg-4	Seg-5	Seg-2	Seg-3	Seg-4	Seg-2	Seg-3
1	Band Range	1 to 36	1 to 36	1 to 36	1 to 36	1 to 36	1 to 36	1 to 37	1 to 37
	Band Count	36	36	36	36	36	36	37	36
	Average Correlation	0.85	0.85	0.85	0.91	0.91	0.91	0.96	0.96
2	Band Range	37 to 102	37 to 80	37 to 79	37 to 204	37 to 106	37 to 81	38 to 103	38 to 72
	Band Count	66	44	43	168	70	45	66	35
	Average Correlation	0.58	0.88	0.89	0.34	0.83	0.9	0.58	0.96
**3**	**Band Range**	**103 to 200**	**81 to 102**	**79 to 102**	**N/A**	**107 to 204**	**82 to 106**	**N/A**	**73 to 103**
	Band Count	98	22	23		98	25		31
	Average Correlation	0.95	0.5	0.51		0.96	0.94		0.95
4	Band Range	N/A	103 to 200	103 to 162	N/A	N/A	107 to 204	N/A	N/A
	Band Count		98	60			98		
	Average Correlation		0.95	0.95			0.96		
**5**	**Band Range**	**N/A**	**N/A**	**163 to 200**	**N/A**	**N/A**	**N/A**	**N/A**	**N/A**
	Band Count			38					
	Average Correlation			0.96					

Next in the preprocessing sequence, mRMR is applied to $Z_{t}$ [56]. The goal of mRMR is to minimize redundancy among selected features and choose a feature subset that is highly significant to the target class. The mutual information between features, denoted as *M*, is used to compute the feature redundancy and relevance in the following ways:

$$Redundancy,R_{d}=\frac{1}{{\left|B_{t}\right|}^{2}}\sum_{b_{i},b_{j}\varepsilon B_{t}}M(b_{i},b_{j})$$ (3)

$$Relevance,R_{e}=\frac{1}{\left|B_{t}\right|}\sum_{b_{i}\varepsilon B_{t}}M(b_{i};c),$$ (4)

where *B*_*t*_ is the extracted feature set acquired after applying SPCA, *b*_*i*_ is the distinct feature and *c* denotes the sample class. By simultaneously maximizing relevance and avoiding redundancy, the mRMR algorithm ranks features. An operator ϕ is used to carry out this procedure, as demonstrated as follows:

$$Max\phi (Relevance,Redundancy)=(R_{e}-R_{d}).$$ (5)

Following the mRMR application, the top 15 features are chosen and split into 3 groups. The top five features compose the highest-priority category. Features rated 6th–10th are in the second group (Moderately Significant), and features ranked 11th–15th are in the third group (Least Significant). $Z_{t}$ represents all of these feature groupings that are produced together, where tϵ[1,2,3].

The HSI data cube is split into tiny, overlapping 3D patches for our multi-branch 3D-2D CNN model. These patches, denoted by $P=s\times s\times B_{t}$, are constructed for the three different reduced datacubes, $Z_{t}$ concentrating on centered spatial positions (*i*,*j*) independently. Every patch encompasses a spatial region s×s and contains every spectral band found in that group. Assuming *X* and *Y* stand for the spatial dimensions of the data cube, the total number of 3D patches, or *n*, is computed as (X−s+1)×(Y−s+1).

### 3.2 Multibranch CNN

Because of its strongly correlated bands, HSI classification needs a large number of training data. It is difficult to get large amounts of ground reference data because of high cost and complexity, which leads to inadequate training data and might result in overfitting and unstable parameters in the model [57]. With their intricate non-linear structure, deep neural networks can extrapolate complex functions, uncover distributed data representations, and effectively describe datasets with fewer training examples than shallow neural networks, which can only extract basic edge information and represent simple functions. The 2D CNN extracts spatial information, neglecting the valuable interband relation among the HSI datacube, whereas over-reliance on 3D CNN may result in an excessively intricate model and perhaps lower classification accuracy [58]. To get over these limitations, this study creates a multi-branch feature fusion model by combining 2D and 3D CNN.

Our model incorporates three branches. Each branch has a combination of 2D and 3D architectures with different kernel sizes, and ReLU serves as the activation function throughout. For each layer in the proposed model, Table 2 gives a detailed explanation of the parameters and distribution. A single 2D CNN layer with 64 filters of 3×3 kernel size and a MaxPool layer of size 2×2 comprise each branch, together with one or more 3D CNN layers. The first branch aims to get deeper semantic properties and consists of three 3D CNN layers with identical 3×3×2 kernel size and 8, 16, and 32 filters, in that order. The MaxPool and 2D CNN layers are situated next to these layers. There are two layers of 3D CNN in the following branch: the first includes eight 5×5×3 3D filters, and the second includes sixteen 3×3×2 filters. Finally, the branch has a single MaxPool layer and a single 2D CNN layer. There are eight 7×7×3 3D filters in the last branch, one 2D CNN, and one MaxPool layer after that. The length, width, and depth of the feature cubes that were taken from each of the three network branches are the same. Combining these attributes results in a cohesive feature representation of the image that accounts for its length and width. As mentioned earlier, the features are split based on their significance using mRMR; the groups with the most significant features are fed into the deep branch, and the groups with the least significant features are fed into the shallow branch. The remaining group of features is carried out to the other branch.

**Table 2:** Configuration of deep neural network used in feature learning procedure.

Branch	Layer	Input Shape	Output Shape	Kernel-size	Filters	Connected to
	Input_3	–	(25,25,5,1)	–	–	
	Conv3D_3_1	(25,25,5,1)	(23,23,4,8)	3×3×2	8	Input_3
1	Conv3D_3_2	(23,23,4,8)	(21,21,3,16)	3×3×2	16	Conv3D_3_1
	Conv3D_3_3	(21,21,3,16)	(19,19,2,32)	3×3×2	32	Conv3D_3_2
	Reshape_3	(19,19,2,32)	(19,19,64)	-	-	Conv3D_3_3
	Conv2D_3	(19,19,64)	(17,17,64)	3×3	64	Reshape_3
	MaxPool_3	(17,17,64)	(8,8,64)	2×2	–	Conv2D_3
	Input_2	–	(25,25,5,1)	–	–	
	Conv3D_2_1	(25,25,5,1)	(21,21,3,8)	5×5×3	8	Input_2
2	Conv3D_2_2	(21,21,3,8)	(19,19,2,16)	3×3×2	16	Conv3D_2_1
	Reshape_2	(19,19,2,16)	(19,19,32)	-	-	Conv3D_2_2
	Conv2D_2	(19,19,32)	(17,17,64)	3×3	64	Reshape_2
	MaxPool_2	(17,17,64)	(8,8,64)	2×2	–	Conv2D_2
	Input_1	–	(25,25,5,1)	–	–	–
	Conv3D_1_1	(25,25,5,1)	(19,19,3,8)	7×7×3	8	Input_1
3	Reshape_1	(19,19,3,8)	(19,19,24)	–	–	Conv3D_1_1
	Conv2D_1	(19,19,24)	(17,17,64)	3×3	64	Reshape_1
	MaxPool_1	(17,17,64)	(8,8,64)	2×2	–	Conv2D_1

The idea behind the structure is to extract features at multilevel. The deep branch contains more 3D layers than the others to extract the most important information, exploiting both the spectral and spatial layout from the top-ranked features in the previous stage. The larger kernel size loses more information than the smaller ones, reducing computations and increasing the number of filters, increasing efficient feature extraction [59]. To extract efficiently with minimal loss of information, the kernel size in the deep branch is kept 3×3×2, and other branches contain filters with larger sizes as they carry less significant features [60]. The kernel size of the 2D CNN is kept smaller (3×3) to exploit the spatial domain without losing much information.

## 4 Experimental analysis

### 4.1 Dataset details

1. Salinas Scene (SC): A collection of images covering 224 spectral bands ranging from 0.4 to 2.45μm was obtained using the AVIRIS sensor in the Salinas Valley, California. With a spatial resolution of 3.7 m, the image has dimensions of 512 ×217 pixels. To lessen distortions caused by water absorption, several bands (108−−112, 154−−167, and 224) were eliminated [34]. Further details on the distribution of pixels among the classes are shown in Table 3.
2. The Pavia University (PU): The Pavia University dataset was obtained by the ROSIS sensor while conducting an airborne survey over Pavia, located in northern Italy. Its spectral bands span 0.43 to 0.86μm and have a spatial resolution of 1.3 m. The image is 610×340 pixels [34]. A thorough analysis of the distribution of pixels among the classes is shown in Table 4.
3. Indian Pines (IP): The Indian Pines dataset was captured over northwest Indiana using the AVIRIS sensor. With 145×145 pixels spread over 224 spectral bands (400–2500 nm), it depicts 16 classes of land cover. Research on remote sensing and HSI categorization frequently makes use of this high-resolution dataset [34]. A thorough class-wise pixel distribution is given in Table 5.

**Table 3:** Number of pixels corresponding to their land cover categories for SC dataset.

No.	Class Labels	Samples
1	Brocoli_green_weeds_1	2009
2	Brocoli_green_weeds_2	3726
3	Fallow	1976
4	Fallow_rough_plow	1294
5	Fallow_smooth	2678
6	Stubble	3959
7	Celery	3579
8	Grapes_untrained	11,271
9	Soil_vineyards_develop	6203
10	Corn_senesced_green_weeds	3278
11	Lettuce_romaine_4wk	1068
12	Lettuce_romaine_5wk	1927
13	Lettuce_romaine_6wk	916
14	Lettuce_romaine_7wk	1070
15	Vineyards_untrained	7268
16	Vineyards_vertical_trellis	1807

**Table 4:** Number of pixels corresponding to their land cover categories for the PU dataset.

No.	Class Labels	Samples
1	Asphalt	6631
2	Meadows	18,649
3	Gravel	2099
4	Trees	3064
5	Painted_metal_sheets	1345
6	Bare_Soil	5029
7	Bitumen	1330
8	Self_Locking_Bricks	3682
9	Shadows	947

**Table 5:** Number of pixels corresponding to their land cover categories for the IP dataset.

No.	Class Labels	Samples
1	Alfalfafa	46
2	Corn-no till	1428
3	Corn-mintill	830
4	Corn	237
5	Grass-pasture	483
6	Grass-trees	730
7	Grass-pasture-mowed	28
8	Hay-windrowed	478
9	Oats	20
10	Soybean-no till	972
11	Soybean-mintill	2455
12	Soybean-clean	593
13	Wheat	205
14	Woods	1265
15	Buildings-Grass-Trees-Drives	386
16	Stone-Steel-Towers	93

### 4.2 Experimental setup

Our experiments involve splitting the data into three categories: training, validation, and testing. For this split, we made a range of ratios, including 20%−80%, 15%−85%, 10%−90%, and 5%−95%. For instance, we randomly chose 10% of the labeled samples for training and validation when utilizing the 10%−90% split, with the remaining 90% acting as the test set. During the CNN training phase, the neural network’s parameters (weights and biases) were adjusted using 80% of the training and validation subset while the other 20% of this subset was exploited to inform our network design selections and to keep an eye out for overfitting.

We start our model by executing SPCA. Each HSI dataset is split into *L* segments, where *L* represents the number of segments and varies from 2 to 5 for different datasets. We make segments based on the average correlation of the bands. The detailed findings of the correlation analysis can be found in Table 1. We use the min-max scaler from Python’s sklearn preprocessing module to normalize the data after SPCA. The following are the hyperparameters of our model: 120 epochs, 64 batch sizes, 0.000001 decay rate, and 0.0001 learning rate. Analysis of classification results led to the determination of these values. From the input volume, we extract 3D patches to provide an even comparison. These patches have *F* spectral characteristics, where *F* is the number of input spectral bands, and have a spatial size of 25×25. For the L2 regularizer, 0.0001 was chosen as the ideal value of L2. We utilized the ReLU activation function everywhere else and the SoftMax activation function in the output layer. In the fully connected layers, the dropout rate is 40%. We conducted our experiments on Kaggle GPU P100.

We utilized six popular quantitative indicators to evaluate the effectiveness of our categorization model: Overall Accuracy (OA): The proportion of properly categorized samples to all test samples, Average Accuracy (AA): The mean of the classification accuracy across classes, Kappa Coefficient: A quantitative indicator of the consistency of qualitative elements, Precision: The ratio of true positive predictions to all positive predictions, Recall: The ratio of true positive prediction to all actual positive values in the dataset, and F1 Score: The harmonic mean of precision and recall. A comparison of the number of trained parameters and the average inference time of the approaches is also introduced in Table 10. In addition to these measures, we observed the accuracy and training loss throughout the training phase.

### 4.3 Impact of segmentation

**Fig 3:**
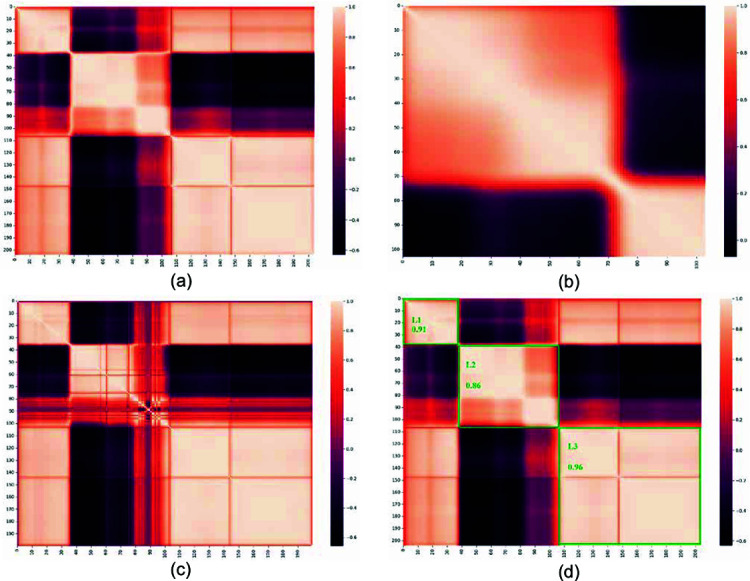
Band-to-band correlation matrix for the datasets, (a) SC, (b) PU, (c) IP, and (d) Band-to-band correlation matrix show the segmentation of SC when L=3.

First, the correlation matrix’s average band-to-band correlation value is used to split each dataset into *L* segments. Three alternative ways are taken to divide the IP and SC datasets, while two ways are considered for PU. Segmentations are named Seg-2, Seg-3, and Seg-4 for SC; Seg-2 and Seg-3 for PU; and Seg-3, Seg-4, and Seg-5 for IP datasets. Fig 3(a)–3(c) shows the band-to-band correlation matrix picture representation for different HSI datasets. For Seg-PCA, correlation values with absolute magnitudes higher than 0.5 are taken into account. As seen in Fig 3(d), this threshold was determined by visually recognizing edges in the band-to-band correlation matrix picture. The figure also shows an example of the segmentation result for the SC HSI dataset. Table 1 displays the segmentation details for the dataset. For each of the three datasets, each segmentation is independently evaluated to find the optimal one. To train and verify the proposed model, we use 15 extracted features, which are found after applying SPCA and mRMR as mentioned earlier. Table 6 displays the classification accuracy results for various segmentations. Except for IP Seg-4 findings, the results show that the Seg-3 or three-segment dataset performs better than Seg-2 and Seg-4. Seg-4 and Seg-5 need more computations, and significant spectral bands might be neglected as we are making more segments and choosing the top features from the segments.

**Table 6:** Outcomes of the proposed model for different segmentations of datasets.

Dataset	Number of Segments	Training Samples	OA	AA	Kappa	Precision	Recall	F1 Score
**SC**	**Seg-2**	**5%**	**99.46**	**99.69**	**99.40**	**94.50**	**94.50**	**94.5**
		10%	99.93	99.89	99.92	99.93	99.93	99.93
		15%	99.97	99.97	99.96	99.97	99.97	99.97
		20%	99.98	99.98	99.98	99.98	99.98	99.98
	Seg-3	5%	**99.87**	**99.89**	**99.89**	**99.87**	**99.87**	**99.87**
		10%	**99.97**	**99.97**	**99.97**	**99.97**	**99.97**	**99.97**
		15%	**99.99**	99.96	99.99	99.99	99.99	99.99
		20%	**100**	**100**	99.99	**100**	**100**	**100**
	Seg-4	5%	99.79	99.82	99.76	99.79	99.79	99.79
		10%	99.96	99.96	99.95	99.96	99.96	99.96
		15%	**99.99**	**99.99**	**99.99**	**99.99**	**99.99**	**99.99**
		20%	99.99	99.99	99.99	99.99	99.99	99.99
**PU**	**Seg-2**	**5%**	**98.78**	**97.77**	**98.39**	**98.79**	**98.78**	**98.78**
		10%	99.36	98.49	99.15	99.36	99.36	99.36
		15%	**99.82**	**99.56**	**99.76**	**99.81**	**99.81**	**99.81**
		20%	**99.94**	**99.93**	**99.93**	**99.95**	**99.95**	**99.94**
	Seg-3	5%	**99.03**	97.73	97.73	**99.03**	**99.02**	**99.02**
		10%	**99.49**	**98.83**	**99.33**	**99.49**	**99.49**	**99.49**
		15%	99.76	99.54	99.68	99.76	99.76	99.76
		20%	99.93	99.89	99.91	99.93	99.93	99.93
**IP**	**Seg-3**	**5%**	**89.81**	**76.21**	**88.32**	**90.06**	**89.81**	**89.5**
		10%	97.02	90.69	96.60	96.85	97.02	96.89
		15%	97.96	95.47	97.67	97.98	97.96	97.94
		20%	99.11	99.28	98.99	99.12	99.11	99.11
	Seg-4	5%	**92.64**	**81.64**	**91.59**	**92.56**	**92.64**	**92.45**
		10%	96.95	**93.46**	96.53	96.96	96.95	96.93
		15%	**98.28**	93.11	**98.04**	**98.3**	**98.28**	**98.25**
		20%	99.11	96.42	98.98	99.11	99.11	99.1
	Seg-5	5%	91.65	73.26	90.44	91.41	91.65	91.18
		10%	**97.4**	90.79	**97.03**	**97.26**	**97.4**	**97.3**
		15%	98.06	**96.95**	97.79	98.08	98.05	98.05
		20%	**99.12**	**99.35**	**99.00**	**99.16**	**99.12**	**99.13**

### 4.4 Result analysis

In this part, the suggested model’s classification performance is methodically investigated using both quantitative and qualitative assessment techniques on the PU, SA, and IP datasets. The study used six metrics—Overall Accuracy (OA), Average Accuracy (AA), Kappa coefficient, Precision, Recall, and F1 score—to evaluate the effectiveness of different classification techniques. These measures were used on several already-existing methods as well as the recently proposed strategy. A comparison analysis is conducted to evaluate the effectiveness of the suggested method against seven top algorithms that are presently considered state-of-the-art. The existing methods include PCA with SVM, SPCA with SVM, 2D-CNN, Fast 3D-CNN, HybridSN, a 2D-3D multibranch fusion model, and IGroupSS-Mamba. All accuracy measurements were stated in percentages (%). Following is a detailed explanation of the state-of-the-art strategies:

1. 2D-CNN [30]: For dimensionality reduction, randomized PCA is used. Two 3×3 convolutional layers with 30 and 90 filter counts, respectively, make up the network. There is also an output layer and two fully connected layers with corresponding dropout rates of 25% and 50%. The classifier uses the SoftMax function, and the activation function used is the ReLU.
2. Fast 3D-CNN [37]: To reduce dimensionality, incremental PCA is applied. With filter counts of 8, 16, 32, and 64, the network is composed of four convolutional layers with dimensions of 3×3×7, 3×3×5, 3×3×3, and 3×3×3, respectively. Then follow the fully connected layers.
3. HybridSN [39]: To reduce dimensionality, the conventional PCA is employed. Three completely linked layers, one 2D convolution layer, and three 3D convolution layers make up this design.
4. 2D-3D multibranch [52]: PCA is used in order to decrease dimensionality. Three separate branches make up this architecture, and they all share the same PCA-extracted features. One 3D and one 2D layer is used in the first branch; two 3D and one 2D layer are used in the second; and three 3D and one 2D layer are used in the third branch. The features from these various branches are then combined and routed via four completely linked layers before arriving at the output layer.
5. IGroupSS-Mamba [61]: To alleviate the Hughes phenomenon, PCA is used initially. The approach consists of the Interval Group S6 Mechanism (IGSM) and multiple stacked Interval Group Spatial-Spectral Blocks (IGSSB).

Tables 7–9 depict the results of all the investigated methods for four categories of training-testing separations. The scenario with the highest performance is bolded. In most circumstances, the suggested approach consistently performs better than other approaches. The three datasets under analysis each have unique features. The PU dataset turns out to be the simplest to categorize, consisting of just nine groups. On the other hand, the SC dataset offers more comprehensive information for categorization, having the most spectral bands and largest spatial dimensions. As a result, on this dataset, most approaches attain greater accuracy rates. The IP dataset poses a special difficulty. Its intricacy causes it to have worse classification accuracy than the other datasets, even if its modest geographical dimension allows it to include 16 categories. When it comes to classification techniques, straight use of PCA with SVM on raw data results in moderate performance. However, preprocessing the data with SPCA before using SVM results in a notable improvement in classification accuracy.

**Table 7:** Comparison of the proposed model with the state of the arts for SC dataset.

Methods	Training Samples	OA	AA	Kappa	Precision	Recall	F1 Score
PCA+SVM	5%	87.54	90.53	86.06	88.00	87.54	86.76
	10%	89.06	92.91	88.05	89.69	89.31	88.66
	15%	89.77	93.51	88.57	90.18	89.77	89.15
	20%	90.04	93.75	88.87	90.42	90.04	89.46
SPCA+SVM	5%	84.78	83.87	82.94	84.31	84.79	82.93
	10%	88.84	92.04	87.53	89.61	88.85	88.18
	15%	89.61	93.03	88.39	90.19	89.61	89.05
	20%	90.35	93.9	89.23	90.81	90.35	89.86
2D CNN	5%	97.06	98.83	96.73	98.90	97.20	98.00
	10%	97.84	99.20	97.60	99.30	97.90	98.60
	15%	98.26	99.36	98.06	99.40	98.30	98.80
	20%	98.37	99.44	98.19	99.50	98.40	98.90
Fast Compact 3D CNN	5%	99.77	98.91	97.51	97.76	97.77	97.76
	10%	99.09	99.54	98.99	99.09	99.09	99.09
	15%	98.52	99.40	98.35	98.63	98.52	98.52
	20%	98.76	97.35	98.62	98.91	98.77	98.73
HybridSN	5%	99.85	99.91	99.83	99.84	99.84	99.84
	10%	99.59	99.76	99.54	99.60	99.60	99.59
	15%	99.97	99.93	99.97	99.97	99.97	99.97
	20%	99.97	99.93	99.97	99.97	99.97	99.97
Multibranch	5%	99.38	99.44	99.31	94.42	94.42	94.42
	10%	99.88	99.91	99.86	99.88	99.88	99.88
	15%	99.92	99.87	99.91	99.92	99.92	99.91
	20%	99.97	99.96	99.97	99.96	99.97	99.97
IGroupSS-Mamba	5%	**99.95**	**99.92**	**99.95**	**99.94**	**99.92**	**99.93**
	10%	99.95	99.94	99.95	99.90	99.93	99.92
	15%	**99.99**	**99.99**	**99.99**	**99.99**	**99.99**	**99.99**
	20%	99.99	99.99	**99.99**	99.98	99.99	99.98
Proposed	5%	99.87	99.89	99.89	99.87	99.87	99.87
	10%	**99.97**	**99.97**	**99.97**	**99.97**	**99.97**	**99.97**
	15%	**99.99**	**99.99**	**99.99**	**99.99**	**99.99**	**99.99**
	20%	**100**	**100**	**99.99**	**100**	**100**	**100**

**Table 8:** Comparison of the proposed model with the state of the arts for the PU dataset.

Methods	Training Samples	OA	AA	Kappa	Precision	Recall	F1 Score
PCA+SVM	5%	78.24	64.28	69.52	74.39	78.24	72.33
	10%	79.39	65.27	71.16	80.10	79.39	73.89
	15%	80.51	66.62	72.87	80.90	80.51	75.65
	20%	81.91	68.43	74.95	81.96	81.91	77.93
SPCA+SVM	5%	81.47	69.54	74.17	80.64	81.47	77.69
	10%	84.09	72.27	87.08	82.56	84.10	81.46
	15%	85.39	73.99	79.98	83.66	85.39	83.11
	20%	86.18	75.29	81.14	84.22	86.18	84.14
2D CNN	5%	95.09	94.19	93.52	95.20	94.70	94.90
	10%	97.74	97.27	97.02	97.80	97.50	97.60
	15%	98.97	98.59	98.63	99.00	98.80	98.90
	20%	99.38	99.07	99.18	98.89	98.50	98.69
Fast Compact 3D CNN	5%	97.34	96.83	96.48	97.37	97.33	97.34
	10%	97.68	97.24	96.92	97.88	97.68	97.69
	15%	99.35	98.9	99.14	99.36	99.35	99.35
	20%	99.46	99.12	99.28	99.46	99.46	99.46
HybridSN	5%	99.01	98.45	98.69	**99.04**	99.01	99.01
	10%	98.75	98.79	98.35	98.80	98.75	98.75
	15%	99.85	99.61	99.80	**99.84**	**99.84**	**99.84**
	20%	99.88	99.76	99.84	99.88	99.88	99.88
Multibranch	5%	98.07	96.45	97.44	98.07	98.07	98.07
	10%	99.07	98.62	98.77	99.09	99.07	99.07
	15%	98.91	97.50	98.55	98.91	98.90	98.90
	20%	99.62	99.54	99.49	99.63	99.62	99.62
IGroupSS-Mamba	5%	**99.62**	98.92	**99.50**	99.03	98.92	98.97
	10%	**99.74**	**99.27**	**99.66**	**99.60**	99.27	99.43
	15%	**99.91**	**99.79**	**99.88**	99.82	99.78	99.81
	20%	**99.96**	**99.95**	**99.95**	**99.97**	**99.95**	**99.96**
Proposed	5%	99.03	**99.73**	97.73	99.03	**99.02**	**99.02**
	10%	99.49	98.83	99.33	99.49	**99.49**	**99.49**
	15%	99.82	99.56	99.76	99.81	99.81	99.81
	20%	99.94	99.93	99.93	99.95	**99.95**	99.94

**Table 9:** Comparison of the proposed model with the state of the arts for the IP dataset.

Methods	Training Samples	OA	AA	Kappa	Precision	Recall	F1 Score
PCA+SVM	5%	56.97	41.81	48.93	58.00	56.97	51.59
	10%	60.85	45.91	53.96	60.27	60.85	56.94
	15%	61.81	51.49	55.10	63.25	61.81	58.24
	20%	64.95	52.43	58.95	65.65	64.95	62.29
SPCA+SVM	5%	61.73	45.12	54.84	63.49	61.73	57.41
	10%	66.24	48.81	60.32	66.57	66.24	62.67
	15%	68.28	51.31	62.79	70.22	68.29	65.05
	20%	70.93	53.74	66.01	72.28	70.93	68.28
2D CNN	5%	64.77	65.55	60.14	67.20	65.10	66.10
	10%	78.53	83.88	75.73	84.50	79.20	81.80
	15%	85.08	91.61	83.11	92.30	85.70	88.90
	20%	87.04	93.60	85.35	94.20	87.80	90.90
Fast Compact 3D CNN	5%	59.55	41.76	52.77	56.38	59.55	56.47
	10%	71.11	51.28	66.54	69.67	71.11	68.64
	15%	69.55	48.97	64.52	68.12	69.55	67.51
	20%	83.39	61.03	80.95	81.54	88.39	82.03
Hybrid SN	5%	87.27	81.41	85.38	87.57	87.27	86.97
	10%	95.72	89.25	95.11	95.65	95.72	95.56
	15%	96.74	96.41	96.28	96.78	96.73	96.71
	20%	99.09	98.35	98.96	99.12	99.09	99.09
Multibranch	5%	82.38	75.52	80.01	83.01	82.37	82.39
	10%	93.08	86.90	92.08	93.01	93.39	92.92
	15%	97.49	95.52	97.13	97.51	97.49	97.48
	20%	98.67	98.35	98.48	98.70	98.67	98.67
IGroupSS-Mamba	5%	**95.15**	**89.60**	**94.45**	**95.71**	**89.60**	92.15
	10%	**98.66**	**98.62**	**98.47**	**97.39**	**98.62**	**97.98**
	15%	98.23	**97.89**	97.98	97.75	97.89	97.92
	20%	**99.33**	96.44	**99.24**	98.82	96.44	97.01
Proposed	5%	92.64	81.64	91.59	92.56	92.64	**92.45**
	10%	97.40	90.79	97.03	97.26	97.40	97.30
	15%	**98.28**	93.11	**98.04**	**98.30**	**98.28**	**98.35**
	20%	99.12	**99.35**	99.00	**99.15**	**99.12**	**99.13**

The 2D-CNN and Fast 3D-CNN approaches exhibit poorer classification accuracy because they do not make full use of spectral information. HybridSN, a straightforward yet efficient method that generates unique features, mixes many 3D CNNs with a 2D CNN. With its distinct kernel design and deeper multibranch feature fusion, the 2D-3D Multibranch technique efficiently avoids overfitting and makes the best use of the combined spectral-spatial information of hyperspectral imaging, leading to high classification performance. The IGroupSS-Mamba also achieves higher accuracy in extracting multiscale and multidirectional global information hierarchically and in groups. The suggested approach makes use of HSI data’s spectral and spatial components. It learns representative and discriminative features by extracting different image characteristics from the primary components of segmented subgroups and fusing them. By minimizing intra-class variations and optimizing interclass differences, this feature fusion strategy eventually improves classification performance.

**Fig 4:**
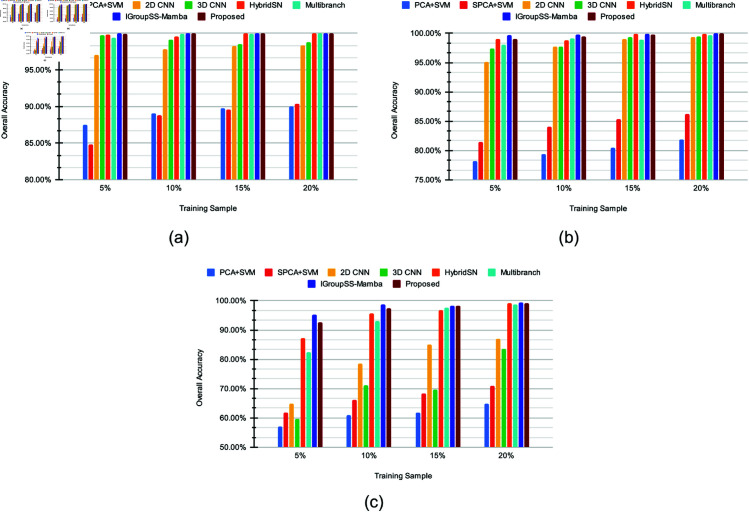
Overall accuracy comparison of (a) SC, (b) PU, and (c) IP datasets for different training samples.

The suggested strategy achieves a perfect OA of 100% for the SC dataset for 20% of training and validation data samples. Despite slightly lagging behind IGroupSS-Mamba for 5% training and validation datasets, the proposed approach outperforms the other test ratios. The suggested strategy performs better than most of the other state-of-the-art approaches, except for providing nearly identical results to IGroupSS-Mamba. For the PU dataset, it achieves an OA of 99.03%, 99.49%, 99.82%, and 99.94% for 5%, 10%, 15%, and 20% training and validation sets, respectively. A similar kind of performance is achieved by the proposed approach for the IP dataset. It surpasses all the other investigated approaches for 15% training-validation sets for the IP dataset. For the other test ratios, it performs closely to IGroupSS-Mamba and outperforms most of the other state-of-the-art approaches, providing 92.64%, 97.40%, 98.28%, and 99.12% for 5%, 10%, 15%, and 20% training-validation sets. Table 10 demonstrates the efficiency of the proposed approach, providing a faster average inference rate for a test sample than most of the investigated techniques. It also predicts 6 to 9.5 times faster than the IGroupSS-Mamba, revealing the proposed technique’s trade-off between accuracy and efficiency. It also maintains an excellent balance between the complexity and performance of the model, though it requires a higher number of parameters than the other investigated methods. Comparing the suggested technique to the HybridSN and 2D-3D Multibranch models under identical spectral characteristics and training sample settings, these findings demonstrate a noteworthy improvement in classification accuracy. Fig. 4 and Tables 7–9 provide the comparison of the investigations graphically and numerically, respectively. It can be observed that our proposed strategy performs better than most of the other state-of-the-art techniques in terms of class-wise accuracy. The detailed comparison is enlisted in Table 11–13. It can be observed that our strategy performs better in most cases for class-wise classifications.

**Table 10:** Computational parameter comparison and analysis.

	Metrics	PCA+SVM	SPCA+SVM	2D CNN	Fast 3D-CNN	HybridSN	Multibranch	IGroupSS-Mamba	Proposed
SC	Params	118117	**43870**	320336	4845696	4845696	33642632	145496	6543400
	Average Inference Time (s)	0.0006	0.0006	0.0002	0.0002	0.0002	0.0007	0.0019	**0.0002**
PU	Params	181469	**66045**	319433	4844793	4844793	33641729	145496	652497
	Average Inference Time (s)	0.0008	0.0008	0.0002	0.0002	0.0002	0.0006	0.0019	**0.0002**
IP	Params	33129	**12379**	320336	4845696	4845696	33642632	145496	6543400
	Average Inference Time (s)	0.0002	0.0002	**0.0002**	0.0003	0.0003	0.0008	0.0018	0.0003

**Table 11:** Class-wise accuracies (%) for the SC dataset with 10% training samples.

Classes	PCA +SVM	SPCA +SVM	2D-CNN	Fast 3D-CNN	HybridSN	2D-3D Multibranch	IGroupSS-Mamba	Proposed
1	98.02	97.86	100	100	100	100	99.78	100
2	99.88	99.91	99.90	100	100	100	100	100
3	86.19	93.77	100	99.86	100	99.97	100	100
4	99.04	99.12	97.07	99.55	99.30	99.60	100	99.92
5	98.72	98.02	98.74	99.77	99.73	99.78	99.54	99.96
6	99.72	99.78	99.14	100	100	100	100	99.99
7	99.88	99.75	100	99.88	100	99.97	99.81	99.98
8	91.08	92.06	100	98.01	99.01	99.82	100	99.99
9	99.46	99.13	78.52	99.92	100	100	100	100
10	91.70	92.41	99.58	99.63	100	99.96	100	99.95
11	87.47	89.32	96.85	99.69	99.85	99.68	100	99.69
12	100	76.82	98.63	99.94	99.83	99.97	100	100
13	98.18	98.43	98.17	99.88	100	100	99.87	100
14	92.07	91.86	98.67	99.79	100	100	100	100
15	47.19	46.30	98.14	97.03	98.50	99.73	100	99.98
16	97.89	98.13	82.50	99.69	100	100	100	100
**OA**	**89.06**	**88.84**	**97.84**	**99.09**	**99.59**	**99.88**	**99.95**	**99.97**
**AA**	**92.91**	**92.04**	**96.62**	**99.54**	**99.76**	**99.91**	**99.94**	**99.97**
**Kappa**	**88.05**	**87.53**	**97.60**	**98.99**	**99.54**	**99.86**	**99.95**	**99.97**

**Table 12:** Class-wise accuracies (%) for the PU dataset with 10% training samples.

Classes	PCA +SVM	SPCA +SVM	2D-CNN	Fast 3D-CNN	HybridSN	2D-3D Multibranch	IGroupSS-Mamba	Proposed
1	93.24	94.27	99.88	95.91	99.68	99.9	100	99.17
2	99.16	99.14	99.88	99.54	99.69	99.67	99.99	99.76
3	1.84	40.39	98.52	93.52	98.35	97.25	99.84	97.45
4	77.53	82.32	98.65	98.28	98.56	98.88	98.01	98.78
5	99.34	99.51	100	100	99.92	100	100	99.92
6	21.62	44.51	99.88	98.79	96.75	99.65	100	99.71
7	0	0	98.7	98.25	99.95	98.65	100	97.42
8	94.74	90.43	98.49	93.29	98.75	98.66	99.88	98.72
9	100	99.88	99.47	97.60	97.46	98.99	95.77	98.56
**OA**	**79.39**	**84.09**	**97.74**	**97.68**	**98.75**	**99.07**	**99.74**	**99.49**
**AA**	**65.27**	**72.27**	**97.27**	**97.24**	**98.79**	**98.62**	**99.27**	**98.83**
**Kappa**	**71.16**	**97.74**	**97.02**	**96.92**	**98.35**	**98.77**	**99.66**	**99.33**

**Table 13:** Class-wise accuracies (%) for the IP dataset with 10% training samples.

Classes	PCA +SVM	SPCA +SVM	2D-CNN	Fast 3D-CNN	HybridSN	2D-3D Multibranch	IGroupSS-Mamba	Proposed
1	5.13	0	88.50	19.89	95.12	92.12	100.00	98.68
2	48.23	56.78	81.81	61.15	94.14	87.57	95.64	95.60
3	23.26	40.63	79.44	30.25	89.01	84.23	99.33	94.90
4	5.12	0.47	89.75	23.28	84.74	74.18	99.53	92.55
5	20.81	21.92	91.51	65.02	98.51	97.65	99.77	95.84
6	94.3	94.60	83.81	88.09	97.52	96.20	99.24	98.40
7	0	0	91.65	0	100	100	100.00	94.66
8	99.09	99.54	86.09	87.90	99.65	100	99.77	100
9	0	0	72.19	0	0	0	100	0
10	37.30	36.96	75.51	57.80	97.55	96.27	97.37	97.53
11	80.84	87.82	86.53	73.22	95.40	93.04	99.91	98.12
12	14.29	40.79	77.88	37.56	91.55	90.70	96.07	96.75
13	95.14	96.76	91.35	93.00	97.95	98.45	98.38	98.48
14	99.02	98.93	93.82	85.20	99.50	98.02	100	99.48
15	31.99	22.19	64.89	50.48	96.12	92.65	100	97.55
16	80.00	83.53	87.36	47.57	91.21	89.36	92.86	94.05
**OA**	**60.85**	**66.24**	**78.53**	**71.11**	**95.72**	**93.08**	**98.66**	**97.40**
**AA**	**45.91**	**48.81**	**83.88**	**51.28**	**89.25**	**86.90**	**98.62**	**90.79**
**Kappa**	**53.96**	**48.81**	**75.73**	**66.54**	**95.11**	**92.08**	**98.47**	**97.03**

**Fig 5:**
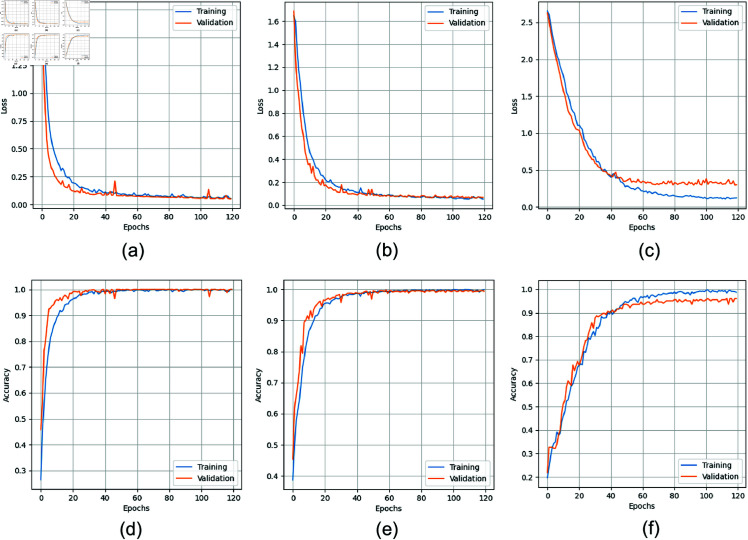
Loss and accuracy curves of all datasets for 10% training samples, (a) SC loss, (b) PU loss, (c) IP loss, (d) SC accuracy, (e) PU accuracy, and (f) IP accuracy.

Fig. 5 shows how the accuracy and loss for the suggested strategies converged across 120 training and validation epochs. The data indicates that our technique reaches stability very rapidly, with convergence occurring at about the 60th epoch for SC and PU and 80th for IP. This quick convergence implies that our method for finding and understanding the underlying patterns in the data is both successful and efficient. Consequently, the approach shows improved classification performance. This fast convergence is important because it indicates that the approach can perform better in classification tasks by swiftly adapting to and representing the key elements of the hyperspectral imaging data. To improve accuracy, our model fuses spectral and spatial data at the pixel level using a multi-branch 3D–2D CNN. For certain hyperspectral image classification applications, our method provides fine-grained information by thoroughly analyzing each pixel’s features.

### 4.5 Ablation study

We conducted an in-depth investigation using three distinct datasets to demonstrate the performance of our proposed model. This study evaluates the performance impact of different configurations of the network components. Our objective was to evaluate our model both with and without segmentation (multibranch) and with and without subgrouping using mRMR. This additional study clarifies the effectiveness of each part of the proposed model, providing useful insights into their roles. The effectiveness of the components along with the comparison is provided in Table 14 and the best results are highlighted in bold. The analysis indicates the superior effectiveness of our proposed segmented approach along with shallow to deep multibranch feature fusion. The study uses mRMR to rank the significance of the extracted feature and then passes it to the branches of the network according to their significance. Removal of this part slightly decreases the classification performance. The same thing happens in the case of using only one branch. Eradicating the segmentation process that includes SPCA and mRMR, the classification accuracy degrades notably. Considering all the investigation, the suggested approach incorporating all of its elements proves to be superior.

**Table 14:** Outcomes of the ablation studies.

Dataset	Approach	Training Sample	OA	AA	Kappa	Precision	Recall	F1 Score
SC	Removal of mRMR	10%	99.90	99.87	99.89	99.90	99.90	99.90
		20%	99.92	99.82	99.91	99.92	99.92	99.92
	Keeping only Deep Branch	10%	99.90	99.91	99.89	99.90	99.90	99.90
		20%	99.94	99.93	99.94	99.95	99.94	99.94
	Removal of Segmentation (Multibranch)	10%	99.88	99.91	99.86	99.88	99.88	99.88
		20%	99.97	99.96	99.97	99.96	99.97	99.97
	Proposed	10%	**99.97**	**99.97**	**99.97**	**99.97**	**99.97**	**99.97**
		20%	**100**	**100**	**99.99**	**100**	**100**	**100**
PU	Removal of mRMR	10%	99.36	98.55	99.15	99.36	99.36	99.35
		20%	99.80	99.66	99.73	99.80	99.80	99.80
	Keeping only Deep Branch	10%	95.59	96.88	94.24	95.97	95.59	95.62
		20%	99.92	99.9	99.9	99.93	99.93	99.92
	Removal of Segmentation (Multibranch)	10%	99.07	98.62	98.77	99.09	99.07	99.07
		20%	99.62	99.54	99.49	99.63	99.62	99.62
	Proposed	10%	**99.49**	**98.83**	**99.33**	**99.49**	**99.49**	**99.49**
		20%	**99.94**	**99.93**	**99.93**	**99.95**	**99.95**	**99.94**
IP	Removal of mRMR	10%	96.95	90.20	96.52	96.81	96.95	96.80
		20%	98.91	98.94	98.76	98.95	98.92	98.92
	Keeping only Deep Branch	10%	96.99	90.53	96.56	96.85	96.99	96.87
		20%	98.95	98.29	98.80	98.72	98.71	98.71
	Removal of Segmentation (Multibranch)	10%	93.08	86.90	92.08	93.01	93.39	92.92
		20%	98.67	98.35	98.48	98.70	98.67	98.67
	Proposed	10%	**97.40**	**90.79**	**97.03**	**97.26**	**97.40**	**97.30**
		20%	**99.12**	**99.35**	**99.00**	**99.15**	**99.12**	**99.13**

## 5 Conclusion and future work

The potential of HSI classification to detect things from a distance has drawn the attention of researchers in remote sensing. Despite a lot of machine learning techniques being tested for HSI classification, they frequently encounter accuracy issues or need a lot of processing power. Deep CNN architectures have portrayed notable performance in this domain. This study demonstrates an innovative approach that combines SPCA and mRMR with a 3D-2D CNN multibranch feature fusion. Our approach creates a multi-branch composition and optimizes CNN-based model generation to meet the current HSI classification issues. The model first split the dataset according to band-to-band correlation. The highly correlated bands are kept together in the same group. SPCA is applied to each of the data segments. Top-ranked features from each group are concatenated. Using the mRMR, the new concatenated dataset is split into three groups according to the features’ significance. The group with the most significant features is carried to the deep branch, and the least significant group is carried to the shallow branch. The remaining features are carried to the branch whose depth is in between shallow and deep. Finally, a fusion of the extracted features is carried out to three fully connected layers and a single softmax output layer. Compared to most of the state-of-the-art techniques, our suggested solution performs better in terms of OA, AA, Kappa, Precision, Recall, and F1 scores.

Although the recommended strategy offers several advantages, it has a few limitations. Specifically, it shows slightly lower accuracy on the IP dataset compared to SC and PU. Additionally, the fully connected layers of the neural network are complex and contain too many parameters. Future work will focus on developing a more streamlined model to enhance generalization and consistency across different datasets.
